# The happiness effect of high-quality development of China’s health and wellness industry: micro-empirical evidence from the Chinese Social Survey 2013–2021

**DOI:** 10.3389/fpubh.2025.1682967

**Published:** 2025-11-14

**Authors:** Song Liu, Wei Zhu

**Affiliations:** School of Economics and Management, Changzhou Institute of Technology, Changzhou, China

**Keywords:** health and wellness industry, high-quality development, happiness, subjective well-being, social security

## Abstract

**Background:**

China has experienced more than 40 years of rapid economic growth and is rapidly entering a deeply aging society. The health needs of Chinese residents have shifted from survival-oriented healthcare to quality-oriented health and wellness services. Promoting the high-quality development of the health and wellness industry is essential for improving national health outcomes and enhancing residents’ overall well-being. While prior research has primarily examined micro-level factors such as older adults care models and medical insurance, empirical studies investigating the happiness effect of industry-level development remain limited.

**Methods:**

This study draws on data from five waves of the Chinese Social Survey (CSS) conducted between 2013 and 2021. A comprehensive indicator system was constructed to measure the high-quality development of China’s health and wellness industry. An ordered logistic regression model was employed for empirical analysis, while also exploring underlying mechanisms and subgroup heterogeneity.

**Results:**

The findings indicate that the high-quality development of the health and wellness industry significantly enhances residents’ happiness. Moreover, the social security systems positively moderate this relationship. Heterogeneity analyses reveal that the happiness-enhancing effect of the high-quality development of the health and wellness industry is more pronounced among female residents, slightly stronger among younger cohorts, notably greater for urban residents, and more significant in eastern China.

**Conclusion:**

This study highlights the positive effect of the high-quality development of China’s health and wellness industry on residents’ happiness, and the positive moderating effect of the social security systems. To further enhance residents’ subjective well-being, it is essential to further improve the quality of development of China’s health and wellness industry. In addition, efforts should be made to deepen the reform of the older adults care security system to better accommodate the increasingly complex care needs of an aging society. Reforming and expanding the medical security system is also vital for improving the accessibility and equity of health services.

## 1 Introduction

In the context of China’s profound demographic transformation, the rising burden of chronic diseases and the deepening implementation of the “Healthy China 2030” strategy have led to a sustained and steady increase in public demand for high-quality health and wellness services. As a vital link connecting healthcare services, older adults care security, and economic development, the health and wellness industry is tasked with the important mission of promoting public health and enhancing overall well-being. The health and wellness industry has evolved from a narrow focus on older adults care into a comprehensive and dynamic industrial ecosystem encompassing health management, leisure tourism, cultural experiences, and smart services. In the first half of 2025, China’s revenue from older adults tourism services and sports and health services grew by 26.2 and 23.9% year-on-year, respectively, reflecting a paradigm shift from “survival-oriented older adults care” to “development-oriented health and wellness.” Digital marketing has brought both impacts and challenges to the development of the health and wellness industry (1). Therefore, through measures such as technological empowerment, resource integration, and institutional design, the health and wellness industry can achieve a high-quality transformation from traditional older adults care to a digitalized, intelligent, and inclusive service system.

The development of the health and wellness industry contributes to healthier, happier, and more dignified lifestyles, thereby enhancing overall happiness. Theoretical perspectives from health psychology, health sociology, and health behavior jointly provide the conceptual foundation for understanding this relationship. These frameworks suggest that the high-quality development of the health and wellness industry enhances individuals’ emotions, sense of meaning, control, and social relationships by shaping social attitudes, rebuilding social networks, empowering individual psychology, and optimizing behavioral environments. According to Self-Determination Theory (SDT), satisfaction of three fundamental psychological needs—autonomy, competence, and relatedness—constitutes the core source of happiness. High-quality health and wellness services can activate these mechanisms by supporting independent living, enhancing physical and cognitive capacities, and fostering social participation. In addition, Social Support Theory (SST) posits that individuals acquire emotional and instrumental resources through social networks, which help them manage stress, maintain physical and mental health, and improve life satisfaction. The detachment from social roles in older adulthood often leads to a “loss of meaning” (2), while the productive aging framework within the health and wellness industry provides opportunities for value reconstruction. Moreover, a robust social security system offers the institutional foundation for residents to fully benefit from the high-quality development of the health and wellness industry.

In the field of happiness research, scholars have conducted many beneficial discussions on conceptual definitions, theoretical frameworks, and influencing factors. Mainstream psychology often equates subjective well-being with “maximizing happiness,” focusing primarily on life satisfaction, positive emotions, and the absence of negative emotions, while overlooking other important dimensions such as meaning, harmony, and spirituality (3). In sociology, researchers have evaluated residents’ happiness from multiple perspectives, including emotional, ethical, and welfare systems (4, 5). Although a linear relationship exists between income and emotional happiness (6, 7), the association between income growth and subjective life quality is more complex, and the relationship between income and happiness is not always consistent (8). With increasing health risks and changes in social roles, the improvement of internet skills and the enhanced social security system directly affect residents’ happiness (9). In addition, recent studies have shown that social relationships, love, and physical activity regulate neurotransmitters and enhance sensory functions, thereby alleviating stress and promoting both health and happiness (10, 11). Similarly, environmental and artistic factors have also been identified as important sources of happiness (12). Furthermore, sensory functions such as hearing and smell have been recognized as physiological bases for social interaction and happiness, and the functional rehabilitation of these senses has been shown to indirectly improve quality of life and happiness (13).

It is worth noting that high-quality economic development, including the cultural industry and health and wellness industry, has spillover effects on residents’ happiness. High-quality economic development promotes happiness by fulfilling residents’ autonomous needs (e.g., learning, self-improvement), capability needs (e.g., goal achievement), and relational needs (e.g., social trust), as well as by providing specialized and diverse cultural products. Furthermore, it contributes to happiness through pathways such as industrial structure upgrading, inclusive total factor productivity (TFP) growth, technological innovation, environmental improvement, and rising living standards (14). In view of the above analysis, this study constructs a comprehensive index system to measure the high-quality development of the health and wellness industry within the Chinese context. Using longitudinal data from the Chinese Social Survey, this study aims to empirically examine the impact of the high-quality development of the health and wellness industry on residents’ happiness, thereby providing evidence-based insights for the formulation of China’s health and wellness policies and for proactive responses to population aging strategies.

## 2 Method

### 2.1 Data sources

The measurement data for the high-quality development of the health and wellness industry was derived from annual statistical data across 31 provinces in China, excluding Hong Kong, Macau, and Taiwan. All data used in this study were obtained from officially published sources. Specifically, data on “Budgetary expenditure on health, culture, tourism and sports, and social security” was sourced from the *China Social Statistical Yearbook*. Data on “Number of people employed in the industry/number of people at the end of the year” was obtained from the *China Population and Employment Statistical Yearbook*. Data on “Insurance coverage rate” and “Number of beds per 1,000 older population” was derived from the *China Civil Affairs Statistical Yearbook*. Data on “Proportion of students with a bachelor’s degree or above” came from the *China Labor Statistical Yearbook*. Data on “Growth rate of health and wellness output value/growth rate of fixed assets investment” were sourced from the *China Industrial Statistical Yearbook*. Data on “Proportion of the tertiary industry in regional GDP” was obtained from the *China Tertiary Industry Statistical Yearbook*. Data on “Number of community service agencies and facilities per 10,000 population” and “Number of beds per 1,000 population in health facilities” were derived from the *China Health Statistical Yearbook*. All other data were obtained from the *China Statistical Yearbook*. Considering data availability, this study measured the level of high-quality development in China’s health and wellness industry from 2012 to 2022. Missing data for individual years were supplemented through interpolation methods.

The data for the dependent variable, moderating variable, and control variables used in this study were obtained from the *Chinese Social Survey* (CSS), a large-scale, nationwide, continuous sampling survey initiated by the Institute of Sociology, Chinese Academy of Social Sciences. The CSS adopts a probability sampling approach and is conducted across 151 counties (districts) and 604 neighborhood (village) committees nationwide. Launched in 2006, the CSS has been carried out approximately every 2 years. To align with the timeframe of the high-quality development data for the health and wellness industry, this study used CSS data from 2013, 2015, 2017, 2019, and 2021.

### 2.2 Variable selection

#### 2.2.1 Dependent variable

The dependent variable in this paper is the individual’s happiness level, which was used in many existing studies (15, 16). It was based on the respondent’s answer to the CSS question: “Do you agree that, overall, I am a happy person?” Response options include “strongly disagree,” “somewhat disagree,” “somewhat agree,” and “strongly agree,” which were assigned values from 1 to 4, respectively. A higher score indicates a higher level of subjective happiness.

#### 2.2.2 Independent variable

The independent variable in this study is the high-quality development of the health and wellness industry. Based on expert interviews, this study constructed an indicator system comprising three dimensions: environmental support for health and wellness, industry competitiveness, and development creativity. This indicator system was used to evaluate the high-quality development of the health and wellness industry across 31 provinces in China, excluding Hong Kong, Macau, and Taiwan. The range normalization method was used to normalize the data, with positive indicators applied when the indicator data tended to be large and negative indicators applied when the indicator data tended to be small. The calculation formula was as: $X_{\mathrm{it}}^{'}=(X_{\mathrm{it}}-\min X_{\mathrm{it}})/(\max X_{\mathrm{it}}-\min X_{\mathrm{it}})$ . The information entropy method was used to calculate the indicator weights. The calculation procedure was as follows. Based on the standardized data, the proportion of indicator *j* for individual *i* in year *t* was calculated as: $Y_{\mathrm{it}}=X_{\mathrm{it}}^{'}/\sum X_{\mathrm{it}}$ . From this, the entropy value was obtained as: $e_{j}=(-1/\ln m)\sum (Y_{\mathrm{it}}\times \ln Y_{\mathrm{it}})$ , and the degree of redundancy as: $d_{j}=1-e_{j}$ , where m represented the number of individuals. The weight of each indicator was then calculated as: $W_{j}=e_{j}/\sum d_{j}$ . Finally, the high-quality development index (HQDI) of the health and wellness industry was calculated as: $\mathrm{HQD}I_{\mathrm{it}}=W_{j}\times X_{\mathrm{it}}^{\prime }$ , with results scaled to a maximum score of 10. The specific indicators were shown in Table 1.

**Table 1:** Measurement index system for high-quality development of the health and wellness industry.

First-level indicators	Secondary indicators	Third-level indicators	Measuring methods	Attribute
Health and wellness environment support	Natural environment	Number of days with good air quality	Number of days when the air quality reached or was better than grade II	+
		Noise equivalent sound level	Equivalent sound level of regional environmental noise	−
		Green coverage ratio	Green coverage rate of built-up areas	+
	Economic environment	General public budget expenditure on health and wellness	Budgetary expenditure on health, culture, tourism & sports, and social security	+
		Development level of the service industry	Proportion of the tertiary industry in regional GDP	+
	Cultural environment	Workforce literacy	Proportion of students with a bachelor’s degree or above	+
		Residents’ awareness of health and wellness	Insurance coverage rate	+
Health and wellness industry competitiveness	Health and wellness resources	Forest health and wellness resources	Land area covered with trees	+
		National nature reserve	Area of national nature reserves	+
	Health and wellness facilities	Community service agencies and facilities	Number of community service agencies and facilities per 10,000 population	+
		Primary medical and health service facilities	Number of beds per 1,000 population in health facilities	+
		Nursing homes and facilities	Number of beds per 1,000 older population	+
		Residential leisure facilities	Per capita green space area	+
	Industrial performance	Labor productivity in the health and wellness industry	Growth rate of health and wellness output value/number of employees	+
		Occupation of health and wellness workers	Number of people employed in the industry/number of people at the end of the year	+
Health and wellness development creativity	Regional health status	Quality of life of population	Life expectancy	+
		Health education and training	Number of health education and training personnel	+
	Contribution of health and wellness industry	GDP contribution rate of health and wellness industry	Proportion of health and wellness output value in regional GDP	+
		Capital productivity of health and wellness industry	Growth rate of health and wellness output value/growth rate of fixed assets investment	+
	Potential of health and wellness market	Per capita consumption of health and wellness	Per capita health and wellness consumption/personal consumption expenditure	+
		Proportion of the older population	Older population dependency ratio	−

#### 2.2.3 Moderating variable

The moderating variable in this study is social security, which is measured by the self-reported social security status of the respondents. Specifically, it was based on responses to two survey questions: (1) “Do you currently have pension insurance or retirement benefits provided by the government?” and (2) “Do you currently have medical insurance or public medical care provided by the government?” For both questions, a response of “yes” was coded as 1, and “no” as 0.

#### 2.2.4 Control variables

This article selected two types of control variables based on previous literature (15, 16). The first type reflected individual characteristics, including gender (female = 0, male = 1), age, ethnicity (ethnic minority = 1, Han = 0), years of education, religious belief (having religious belief = 1, no religious belief = 0), political status (member of the Communist Party of China (CPC) = 1, others = 0), household registration (also known as hukou, urban = 1, rural = 0), place of residence (urban = 1, rural = 0), employment status (employed = 1, unemployed = 0), marital status (married or cohabiting = 1, single = 0), personal income (natural logarithm of annual income), socioeconomic status (lower level = 1, lower-middle level = 2, middle level = 3, upper-middle level = 4, upper level = 5). The second type reflected family characteristics, including family size (number of family members), housing units (number of housing units owned by the family), and per capita household income (natural logarithm of annual per capita household income).

#### 2.2.5 Descriptive statistics

Table 2 presents the descriptive statistics of the variables. The average happiness score among residents was 3.137, suggesting a relatively high level of self-reported happiness. The standard deviation of the HQDI of the health and wellness industry was 0.559, indicating notable regional disparities. Regarding demographic characteristics, the average age of respondents was 46.363 years, with relatively balanced distributions in gender, religious belief, household registration, and place of residence. The average years of education were 9.107 years. Most respondents were married and employed. Respondents generally perceived their socioeconomic status to be close to the middle level within their local context.

**Table 2:** Descriptive statistics of the variables.

Variable	N	Mean	Std. Dev.	Min	Max
Happiness	18,228	3.138	0.712	1	4
HQDI	18,228	4.045	0.559	2.625	5.282
Pension security	18,082	0.559	0.497	0	1
Medical security	18,069	0.825	0.382	0	1
Gender	18,228	0.446	0.497	0	1
Age	18,228	46.360	14.051	18	70
Ethnicity	18,228	0.289	0.453	0	1
Years of education	18,204	9.110	4.358	0	18
Religious belief	18,228	0.553	0.497	0	1
Political status	18,228	0.106	0.308	0	1
Household registration	18,228	0.335	0.472	0	1
Place of residence	18,228	0.565	0.496	0	1
Employment status	18,228	0.638	0.481	0	1
Marital status	18,125	0.823	0.382	0	1
Personal income	17,586	8.384	3.523	0	15.096
Socioeconomic status	18,149	2.284	0.911	1	5
Family size	18,228	4.538	2.047	1	25
Housing units	17,710	1.203	0.571	0	7
Per capita household income	17,148	9.243	1.481	−2.398	14.112

### 2.3 Model

According to the Health Capital Theory proposed by American economist Michael Grossman, health is not a fleeting consumption good but a durable form of capital. The health and wellness industry can essentially be regarded as a systematic, specialized, and large-scale platform for health capital investment. Its high-quality development can significantly enhance the efficiency and inclusivity of such investments, thereby contributing to an overall improvement in residents’ happiness. Since the dependent variables, happiness, was an ordered categorical variable, the ordered logistic regression model was adopted to investigate the impact of high-quality development of the health and wellness industry on happiness. The benchmark model is shown in Equation 1:


$${\text{Happiness}}_{\mathrm{it}}=\alpha +\beta {\text{HQDI}}_{\mathrm{it}}+\sum_{i}^{j}\gamma_{j}X_{\mathrm{jit}}+\varepsilon_{\mathrm{it}}$$ (1)

Where $\text{Happines}s_{\mathrm{it}}$ represents the happiness of individual *i* in year *t*; $\mathrm{HQD}I_{\mathrm{it}}$ represents the high-quality development level of the health and wellness industry in the province where individual *i* resides in year *t*; $X_{\mathrm{jit}}$ represents a series of control variables (see Table 2 for details); α 、 β 、 $\gamma_{j}$ represent the parameters to be estimated; $\varepsilon_{\mathrm{it}}$ represents the random error term. The ordered logistic regression model can output the odds ratio OR , $\text{OR}=e^{\left(\beta \right)}$ , which measures the ratio of the probability of changes in the dependent variable after a unit change in the independent variable.

Given that the social security system can provide stable economic support for residents and lower the costs associated with medical and older adults care services, it may potentially enhance the happiness effect of the high-quality development of the health and wellness industry. In order to test whether there is a moderating effect of social security on the relationship between the high-quality development of the health and wellness industry and residents’ happiness, this paper constructed the following moderating effect model:


$$\begin{array}{l} \text{Happines}s_{\mathrm{it}}=\alpha +\beta {\text{HQDI}}_{\mathrm{it}}+\theta M_{\mathrm{it}}+\delta {\text{HQDI}}_{\mathrm{it}}\times M_{\mathrm{it}}+\\ \;\sum_{i}^{j}\gamma_{j}X_{\mathrm{jit}}+\varepsilon_{i,t} \end{array}$$ (2)

In Equation 2, $M_{\mathrm{it}}$ represents the moderating variables, including pension security and medical security; $\mathrm{HQD}I_{\mathrm{it}}\times M_{\mathrm{it}}$ represents the interaction term of the high-quality development of the health and wellness industry and social security; the meanings of the remaining variables are the same as in Equation 1. Here, the focus is on the statistical significance and sign of the coefficient δ .

## 3 Results

### 3.1 Benchmark regression results

Based on controlling for individual and family characteristic variables, this study examined the impact of high-quality development of the health and wellness industry on residents’ happiness, as shown in Table 3. The results of Model 1 indicate that the high-quality development of the health and wellness industry has a significant positive effect on residents’ happiness, contributing to its improvement. Model 2 reports the regression results after gradually removing control variables with *p*-values above 10%. The positive effect of high-quality development remains statistically significant. In Model 2, the regression coefficient (*β*) of the HQDI is 0.269, with an odds ratio (*OR*) of approximately 1.308. This implies that a one-unit increase in the HQDI corresponds to a 30.8% increase in the probability of residents reporting a higher level of happiness.

**Table 3:** Benchmark regression results.

Variable	Model 1		Model 2	
	*β* (SE)	*OR* (95%CI)	*β* (SE)	*OR* (95%CI)
HQDI	0.269 (0.077)^***^	1.309 (1.125–1.522)^***^	0.269 (0.074)^***^	1.308 (1.131–1.513)^***^
Gender	−0.064 (0.035)^+^	0.938 (0.876–1.003)^+^	−0.056 (0.032)^+^	0.945 (0.888–1.007)^+^
Age	−0.080 (0.009)^***^	0.923 (0.906–0.940)^***^	−0.077 (0.009)^***^	0.926 (0.911–0.942)^***^
Quadratic term of age	0.001 (0.000)^***^	1.001 (1.001–1.001)^***^	0.001 (0.000)^***^	1.001 (1.001–1.001)^***^
Ethnicity	0.040 (0.074)	1.041 (0.900–1.204)		
Years of education	−0.003 (0.005)	0.997 (0.986–1.007)		
Religious belief	−0.012 (0.083)	0.988 (0.840–1.162)		
Political status	0.251 (0.053)^***^	1.285 (1.159–1.425)^***^	0.272 (0.049)^***^	1.313 (1.193–1.445)^***^
Household registration	0.043 (0.043)	1.044 (0.960–1.136)		
Place of residence	0.058 (0.041)	1.060 (0.978–1.148)	0.077 (0.036)^*^	1.080 (1.008–1.158)^*^
Employment status	−0.023 (0.043)	0.977 (0.899–1.063)		
Marital status	0.481 (0.055)^***^	1.618 (1.452–1.803)^***^	0.481 (0.053)^***^	1.597 (1.438–1.772)^***^
Personal income	0.007 (0.006)	1.007 (0.996–1.019)		
Socioeconomic status	0.472 (0.017)^***^	1.603 (1.549–1.659)^***^	0.470 (0.017)^***^	1.600 (1.548–1.653)^***^
Family size	0.032 (0.009)^**^	1.033 (1.014–1.052)^**^	0.038 (0.009)^***^	1.039 (1.021–1.057)^***^
Housing units	0.055 (0.030)^+^	1.056 (0.997–1.119)		
Per capita household income	0.069 (0.015)^***^	1.072 (1.041–1.104)^***^	0.076 (0.014)^***^	1.079 (1.050–1.108)^***^
Year fixed effect	Yes		Yes	
Provincial fixed effect	Yes		Yes	
*N*	16,144		17,418	
Pseudo *R*^2^	0.084		0.082	

*β* is the regression coefficient, SE is the robust standard error, OR is the odds ratio, and 95%CI is the 95% confidence interval. +, *, **, and *** indicate *p* < 0.1, *p* < 0.05, *p* < 0.01, and *p* < 0.001, respectively. Yes means the corresponding variables are controlled in the regression. The same below.

Specifically, among the control variables, gender is significantly negatively associated with happiness, suggesting that men report lower levels of happiness than women. The coefficient of age is negative, while the coefficient of the quadratic term of age is positive, indicating a U-shaped relationship between age and happiness. That is, happiness initially decreases with age and then increases. The turning point occurs at approximately 38.5 years old. Political status, place of residence, marital status, socioeconomic status, are all significantly positively associated with happiness. This suggests that, on average, members of the CPC, individuals who live in urban, are married or cohabiting, and those with higher self-perceived socioeconomic status report higher levels of happiness. Regarding family characteristics, both family size and per capita household income are significantly positively associated with happiness, indicating that individuals living in larger households and with higher per capita income tend to be happier.

### 3.2 Robustness tests

In this section, ordered probit (OP) model, ordinary least squares (OLS) model, and generalized ordered logistic (GOL) model were used to test the robustness of the positive effect of the high-quality development of the health and wellness industry on residents’ happiness. The results of robustness test reported in Table 4 (see Supplementary Table S1 for details) indicate that the results from the OP and OLS models are consistent with the benchmark regression, with the high-quality development of the health and wellness industry having a significant positive effect on residents’ happiness at the 1% level.

**Table 4:** Results of robustness test.

Variable	(1) OP	(2) OLS	(3) GOL		
			1 vs 2, 3, 4	1, 2 vs 3, 4	1, 2, 3 vs 4
HQDI	0.142 (0.030)^***^	0.071 (0.017)^***^	0.089 (0.150)	0.157 (0.079)^*^	0.327 (0.087)^***^
Control variables	Yes	Yes	Yes	Yes	Yes
Year fixed effect	Yes	Yes	Yes	Yes	Yes
Provincial fixed effects	Yes	Yes	Yes	Yes	Yes
*N*	17,418	17,418	17,418		
Pseudo *R*^2^	0.081	0.147	0.111		

The GOL model relaxes the parallel lines assumption of the ordered logistic (OL) model and allows for heterogeneity in the effects of independent variables at different levels of the outcome. The original ordinal variable of happiness is collapsed into two categories, such as category 1 versus categories 2, 3, 4. Results in column (3) show that the coefficients of HQDI differ across regressions, so that the parallel lines assumption of OL model is not met. This means the impact of high-quality development varies in magnitude across the range of happiness.

### 3.3 Moderating effect test

Following the moderating effect test procedure proposed by Aiken and West (17), this study introduced interaction terms between social security variables (pension and medical security) and the high-quality development index of the health and wellness industry into the benchmark model to examine the moderating role of social security. Column (1) of Table 5 (see Supplementary Table S2 for details) reports the results of the benchmark regression analysis. Columns (2) and (3) of Table 5 present the results after including the interaction terms between pension security, medical security, and the high-quality development index. The coefficients of the interaction terms are statistically significant at the 10% level and 5% level, respectively, indicating that social security positively moderates the relationship between high-quality development of the health and wellness industry and residents’ happiness.

**Table 5:** Results of moderating effect test.

Variable	(1)	(2)	(3)
HQDI	0.269 (0.074)^***^	0.249 (0.085)^**^	0.241 (0.071)^**^
Pension security		0.048 (0.036)	
Pension security × HQDI		0.020 (0.017)^+^	
Medical security			0.082 (0.048)^+^
Medical securityl × HQDI			0.028 (0.012)^*^
Control variables	Yes	Yes	Yes
Year fixed effect	Yes	Yes	Yes
Provincial fixed effect	Yes	Yes	Yes
*N*	17,418	17,290	17,274
Pseudo *R*^2^	0.082	0.083	0.083

### 3.4 Heterogeneity analysis

The above analysis indicates that the high-quality development of the health and wellness industry significantly enhances residents’ happiness. However, this effect may vary across different demographic groups. Therefore, this study explored the heterogeneity of the impact from four dimensions: gender, age, urban–rural status, and region. The results are presented in Table 6 (see Supplementary Table S3 for details).

**Table 6:** Results of heterogeneity analysis.

Variable	(1) Male	(2) Female	(3) Younger	(4) Older
HQDI	0.191 (0.112)^+^	0.340 (0.104)^**^	0.294 (0.111)^**^	0.239 (0.109)^*^
Control variable	Yes	Yes	Yes	Yes
Year fixed effect	Yes	Yes	Yes	Yes
Provincial fixed effect	Yes	Yes	Yes	Yes
*N*	7,826	9,592	8,609	8,809
Pseudo *R*^2^	0.080	0.082	0.067	0.098

Variable	(5) Urban	(6) Rural	(7) East	(8) Midwest
HQDI	0.280 (0.116)^*^	0.189 (0.106)^+^	0.346 (0.120)^**^	0.163 (0.101)^+^
Control variable	Yes	Yes	Yes	Yes
Year fixed effect	Yes	Yes	Yes	Yes
Provincial fixed effect	Yes	Yes	Yes	Yes
*N*	9,874	7,544	6,037	11,381
Pseudo *R*^2^	0.074	0.097	0.076	0.085

First, the results indicate that the impact of the high-quality development of the health and wellness industry on residents’ happiness varies by gender, with a generally stronger effect observed among female residents. Second, the sample is divided into younger and older groups based on the median age of 47. Regression results show that the effect is slightly greater for younger residents. Third, significant urban–rural disparities are also observed, with urban residents experiencing a more pronounced increase in happiness. Fourth, according to the geographic division of China, the sample is divided into eastern and mid-western regions. The regression results suggest that the positive effect of the high-quality development of the health and wellness industry on residents’ happiness is more substantial in the eastern region.

## 4 Discussion

Driven by the dual forces of an aging population and rising demand for health-related consumption, China’s health and wellness industry has entered a critical stage of quality enhancement and capacity expansion. In order to investigate the impact of the high-quality development of the health and wellness industry on residents’ happiness, this study constructed an indicator system based on three dimensions: environmental support, industry competitiveness, and development creativity, to measure the high-quality development index of the health and wellness industry across China. Empirical results indicate that the high-quality development of the health and wellness industry significantly enhances residents’ happiness, consistent with Wei et al. (14). This finding further confirms the vital role of industrial upgrading in meeting people’s aspirations for a better life (18).

Further analysis reveals that social security systems play a positive moderating role in this relationship. On the one hand, the high-quality development of the health and wellness industry can drive the innovation of the pension security system. Smart healthcare and wellness significantly improve residents’ satisfaction with participation in and benefits from pension security (8). Enhancements in the pension security system amplifies the positive impact of the upgrading of the healthcare and wellness industry on residents’ happiness (19). In addition, both family-based and social pension models contribute positively to residents’ well-being, mainly through the provision of emotional support and medical security, respectively. It is necessary to accelerate the establishment of a multi-level pension insurance system, comprehensively promote and optimize the long-term care insurance system, and clearly include eligible home-based, community-based, and institutional care, as well as assistive device services, within the payment scope.

On the other hand, medical insurance significantly strengthens the role of health and wellness industry upgrading in promoting residents’ happiness by reducing health-related uncertainties and improving service accessibility. In addition, targeted financial support for vulnerable groups can bring substantial and lasting improvements in happiness (20). Similarly, greater investment in health insurance reform effectively reduces poverty risks caused by illness (21), alleviates concerns about access to health services, and enhances overall well-being. AI technology also improves accessibility and affordability of healthcare while reducing the stigma associated with mental health (22). These advancements provide a strong rationale for vigorously advancing the integration of medical and older adults care, encourage the establishment of medical facilities within older adults care institutions or partnerships with hospitals, and build an efficient two-way referral system. At the same time, extending medical services to communities and households—through family doctor contracts and home care—can help channel medical and older adults care resources to the grassroots level. In addition, the development of smart healthcare should be encouraged to build an integrated and continuous health and wellness service system.

This study further examines the heterogeneous impact of the high-quality development of the health and wellness industry on the happiness of different groups. First, this paper finds that different gender and age groups exhibit distinct needs for health and wellness services based on their life-cycle stages, social roles, and living environments. The results regarding gender differences suggest that the high-quality development of the health and wellness industry has a more pronounced effect on women’s happiness. This may be because the high-quality development of the health and wellness industry helps alleviate women’s caregiving burden and provides more flexible employment opportunities (23), thereby enhancing their happiness. The impact on younger residents is also found to be slightly greater. One possible explanation is that the high-quality development of the health and wellness industry can better satisfy younger individuals’ pursuit of a high-quality, healthy lifestyle and spiritual fulfillment, help alleviate their anxiety about the future, and partially relieve the burden of parental care. Second, findings on urban–rural disparities indicate that the happiness effect is more pronounced among urban residents. Although earlier studies suggested that the happiness effects of smart healthcare are more pronounced among less-educated and rural residents (24), urban residents—who often face spatial constraints, time scarcity, and environmental pressures—are also highly sensitive to the well-being improvements brought by the high-quality development of the health and wellness industry. Third, the happiness effect of the high-quality development of the health and wellness industry exhibits substantial regional variation. In eastern China, the accumulation of financial capital, advanced digital infrastructure, and well-developed health and wellness services have fostered relatively strong demand for health and wellness consumption, further contributing to increased happiness among residents. Therefore, it is essential to promote the transformation and upgrading of the health and wellness industry and to develop differentiated strategies tailored to the needs of specific groups and regions—particularly younger individuals, women, and urban residents. The eastern region should take the lead in exploring innovative models for high-quality industrial development, thereby maximizing its social benefits in enhancing residents’ happiness.

This study still has several limitations. First, it does not fully address the health and wellness challenges faced by structurally vulnerable groups such as older adults migrants and individuals with disabilities, limiting the generalizability of the findings. Second, due to data availability constraints, this study measured social security using a binary variable, without capturing its multidimensional aspects such as coverage, benefit levels, accessibility, and sustainability. Third, this paper failed to obtain sufficient data to calculate the high-quality development index of the health and wellness industry at the prefecture level or county level, which may affect the accuracy of the analysis results. Furthermore, although the indicator system for assessing the high-quality development of the healthcare and wellness industry includes 21 measurement indicators, it may still not fully represent the overall development level. Some indicators may also conceptually overlap with control or moderating variables, which could introduce potential multicollinearity issues. These should be considered in future research.

## 5 Conclusion

Based on five waves of data from the Chinese Social Survey (CSS), this study reveals a significant positive effect of the high-quality development of China’s health and wellness industry on residents’ happiness. In other words, promoting the high-quality development of the healthcare and wellness industry serves as an effective pathway to enhance residents’ well-being. Moreover, a sound social security system can improve access to health and wellness services, amplify the welfare effects of industrial development, and therefore the social benefits of the health and wellness industry require a sound social security system as support. Furthermore, differentiated development strategies should be formulated according to the characteristics of different groups and regions—particularly addressing the needs of younger individuals, women, and urban residents in eastern China—to maximize the industry’s contribution to improving residents’ happiness.

## Data Availability

Publicly available datasets were analyzed in this study. This data can be found here: http://css.cssn.cn/css_sy/.
