# Supplementary material for: The happiness effect of high-quality development of China’s health and wellness industry: micro-empirical evidence from the Chinese Social Survey 2013–2021
Source: Front Public Health. 2025 Nov 14;13:1682967. doi: 10.3389/fpubh.2025.1682967 (PMC12661991; doi:10.3389/fpubh.2025.1682967)
Supplement: Supplementary file 1 [file Table_1.docx]

Supplementary material

Supplementary Table S1 Results of robustness test.

| **Variable** | **(1) OP** | **(2) OLS** | **(3) GOL** | | |
| --- | --- | --- | --- | --- | --- |
|  |  |  | **1 vs 2,3,4** | **1,2 vs 3,4** | **1,2,3 vs 4** |
| HQDI | 0.142 (0.030)^***^ | 0.071 (0.017)^***^ | 0.089 (0.150) | 0.157 (0.079)^*^ | 0.327 (0.087)^***^ |
| Gender | -0.038 (0.020)^+^ | -0.023 (0.011)^*^ | -0.181 (0.098)^*^ | -0.128 (0.054)^*^ | -0.041 (0.040) |
| Age | -0.052 (0.005)^***^ | -0.031 (0.003)^***^ | -0.149 (0.027)^***^ | -0.138 (0.015)^***^ | -0.068 (0.011) ^***^ |
| Quadratic term of age | 0.001 (0.000)^***^ | 0.000 (0.000)^***^ | 0.002 (0.000)^***^ | 0.001 (0.000)^***^ | 0.001 (0.000)^***^ |
| Ethnicity | 0.032(0.041) | 0.020 (0.024) | 0.212 (0.153) | 0.077 (0.095) | -0.036 (0.075) |
| Years of education | -0.001 (0.003) | 0.001 (0.002) | 0.040 (0.014)^**^ | 0.041 (0.008)^***^ | -0.028 (0.006)^***^ |
| Religious belief | -0.013 (0.046) | -0.011 (0.026) | -0.231 (0.193) | -0.278 (0.104)^**^ | 0.088 (0.072) |
| Political status | 0.137 (0.031)^***^ | 0.072 (0.017)^***^ | 0.478 (0.235)^**^ | 0.336 (0.114)^**^ | 0.281 (0.063)^***^ |
| Household registration | 0.014 (0.025) | 0.007 (0.014) | 0.089 (0.134) | 0.104 (0.072) | 0.007 (0.050) |
| Place of residence | 0.027 (0.023) | 0.018 (0.013) | 0.075 (0.112) | 0.154 (0.061)^*^ | 0.009 (0.046) |
| Employment status | -0.012 (0.024) | -0.004 (0.014) | 0.135 (0.116) | 0.037 (0.064) | -0.038 (0.047) |
| Marital status | 0.251 (0.031)^***^ | 0.160 (0.019)^***^ | 0.831 (0.117)^***^ | 0.617 (0.072)^***^ | 0.269 (0.061)^***^ |
| Personal income | 0.002 (0.003) | 0.001 (0.002) | -0.026 (0.018) | 0.009 (0.009) | 0.004 (0.007) |
| Socioeconomic status | 0.278 (0.011)^***^ | 0.162 (0.006)^***^ | 0.863 (0.062)^***^ | 0.691 (0.031)^***^ | 0.392 (0.022)^***^ |
| Family size | 0.022 (0.005)^***^ | 0.013 (0.003)^***^ | 0.054 (0.025)^*^ | 0.045 (0.014)^***^ | 0.025 (0.010)^*^ |
| Housing units | 0.016 (0.017) | 0.007 (0.010) | -0.048 (0.087) | 0.052 (0.051) | 0.059 (0.034)^+^ |
| Per capita household income | 0.040 (0.008)^***^ | 0.027 (0.005)^***^ | 0.142 (0.029)^***^ | 0.105 (0.017)^***^ | 0.025 (0.015)^+^ |
| Year fixed effect | Yes | Yes | Yes | Yes | Yes |
| Provincial fixed effects | Yes | Yes | Yes | Yes | Yes |
| N | 17,418 | 17,418 | 17,418 | | |
| Pseudo *R*^2^ | 0.081 | 0.147 | 0.111 | | |

Note: +, *, **, and *** indicate *P* <0.1, *P* <0.05, *P* <0.01, and *P* <0.001 respectively. Yes means the corresponding variables are controlled in the regression.

Supplementary Table S2 Results of moderating effect test.

| **Variable** | **（1）** | **（2）** | **（3）** |
| --- | --- | --- | --- |
| HQDI | 0.269 (0.074)^***^ | 0.249 (0.085)^**^ | 0.241 (0.071)^**^ |
| Pension security |  | 0.048 (0.036) |  |
| Pension security 🞨 HQDI |  | 0.020 (0.017)^+^ |  |
| Medical security |  |  | 0.082 (0.048)^+^ |
| Medical securityl 🞨 HQDI |  |  | 0.028 (0.012)^*^ |
| Gender | -0.064 (0.035)^+^ | -0.063 (0.035)^+^ | -0.063 (0.035)^+^ |
| Age | -0.080 (0.009)^***^ | -0.091 (0.009)^***^ | -0.091 (0.009)^***^ |
| Quadratic term of age | 0.001 (0.000)^***^ | 0.001 (0.000)^***^ | 0.001 (0.000)^***^ |
| Ethnicity | 0.040 (0.074) | 0.032 (0.075) | 0.031 (0.075) |
| Years of education | -0.003 (0.005) | -0.005 (0.005) | -0.005 (0.005) |
| Religious belief | -0.012 (0.083) | 0.005 (0.083) | 0.004 (0.083) |
| Political status | 0.251 (0.053)^***^ | 0.226 (0.053)^***^ | 0.226 (0.053)^***^ |
| Household registration | 0.043 (0.043) | 0.017 (0.043) | 0.016 (0.043) |
| Place of residence | 0.058 (0.041) | 0.048 (0.041) | 0.049 (0.041) |
| Employment status | -0.023 (0.043) | -0.023 (0.042) | -0.023 (0.042) |
| Marital status | 0.481 (0.055)^***^ | 0.437 (0.055)^***^ | 0.439 (0.055)^***^ |
| Personal income | 0.007 (0.006) | 0.005 (0.006) | 0.005 (0.006) |
| Socioeconomic status | 0.472 (0.017)^***^ | 0.482 (0.020)^***^ | 0.482 (0.020)^***^ |
| Family size | 0.032 (0.009)^**^ | 0.039 (0.009)^***^ | 0.039 (0.009)^***^ |
| Housing units | 0.055 (0.030)^+^ | 0.031 (0.030) | 0.032 (0.030) |
| Per capita household income | 0.069 (0.015)^***^ | 0.070 (0.015)^***^ | 0.069 (0.015)^***^ |
| Year fixed effect | Yes | Yes | Yes |
| Provincial fixed effect | Yes | Yes | Yes |
| N | 17,418 | 17,290 | 17,274 |
| Pseudo *R*^2^ | 0.082 | 0.083 | 0.083 |

Note: +, *, **, and *** indicate *P* <0.1, *P* <0.05, *P* <0.01, and *P* <0.001 respectively. Yes means the corresponding variables are controlled in the regression.

Supplementary Table S3 Results of heterogeneity analysis.

| **Variable** | 1. **Male** | **(2) Female** | **(3) Younger** | **(4) Older** |
| --- | --- | --- | --- | --- |
| HQDI | 0.191 (0.112)^+^ | 0.340 (0.104)^**^ | 0.294 (0.111)^**^ | 0.239 (0.109)^*^ |
| Gender |  |  | -0.124(0.049)^*^ | -0.029 (0.050) |
| Age | -0.103 (0.014)^***^ | -0.083 (0.013)^***^ | -0.162 (0.029)^***^ | -0.192 (0.075)^*^ |
| Quadratic term of age | 0.001 (0.000)^***^ | 0.001 (0.000)^***^ | 0.002 (0.000)^***^ | 0.002 (0.001)^**^ |
| Ethnicity | 0.004 (0.109) | 0.046 (0.105) | 0.077 (0.108) | -0.023 (0.107) |
| Years of education | -0.008 (0.009) | -0.005 (0.007) | -0.002 (0.009) | -0.006 (0.007) |
| Religious belief | -0.050 (0.136) | 0.044 (0.105) | -0.039 (0.119) | 0.045 (0.115) |
| Political status | 0.232 (0.067)^***^ | 0.215 (0.088)^*^ | 0.220 (0.076)^***^ | 0.200 (0.073)^**^ |
| Household registration | 0.094 (0.064) | -0.033 (0.058) | 0.031 (0.058) | 0.004 (0.066) |
| Place of residence | 0.005 (0.061) | 0.070 (0.055) | -0.001 (0.058) | 0.095 (0.058) |
| Employment status | -0.002 (0.068) | -0.039 (0.055) | -0.008 (0.071) | -0.021 (0.056) |
| Marital status | 0.582 (0.086) | 0.303 (0.075)^***^ | 0.490 (0.084)^***^ | 0.446 (0.079)^***^ |
| Personal income | 0.003 (0.012) | 0.006 (0.007) | 0.011 (0.008) | 0.002 (0.010) |
| Socioeconomic status | 0.442 (0.030)^***^ | 0.518 (0.027)^***^ | 0.375 (0.029) ^***^ | 0.552 (0.027)^***^ |
| Family size | 0.042 (0.014)^**^ | 0.035 (0.013)^**^ | 0.022 (0.017) | 0.043 (0.011)^***^ |
| Housing units | 0.027 (0.043) | 0.036 (0.042) | 0.010 (0.041) | 0.031(0.044) |
| Per capita household income | 0.057 (0.022)^***^ | 0.085 (0.022)^***^ | 0.075 (0.026)^**^ | 0.064(0.018)^***^ |
| Year fixed effect | Yes | Yes | Yes | Yes |
| Provincial fixed effect | Yes | Yes | Yes | Yes |
| N | 7,826 | 9,592 | 8,609 | 8,809 |
| Pseudo *R*^2^ | 0.080 | 0.082 | 0.067 | 0.098 |
| **Variable** | **(5) Urban** | **(6) Rural** | **(7) East** | **(8) Midwest** |
| HQDI | 0.280 (0.116)^*^ | 0.189 (0.106)^+^ | 0.346 (0.120)^**^ | 0.163 (0.101)^+^ |
| Gender | -0.079 (0.046)^+^ | -0.083 (0.053) | -0.114 (0.060)^+^ | -0.033 (0.042) |
| Age | -0.099 (0.013)^***^ | -0.086 (0.014)^***^ | -0.094 (0.017)^***^ | -0.090 (0.011)^***^ |
| Quadratic term of age | 0.001 (0.000)^***^ | 0.001 (0.000)^***^ | 0.001 (0.000)^***^ | 0.001 (0.000)^***^ |
| Ethnicity | 0.096 (0.111) | -0.027 (0.102) | 0.072 (0.215) | 0.025 (0.080) |
| Years of education | -0.019 (0.008)^*^ | 0.011 (0.008) | -0.018 (0.010)^+^ | 0.000 (0.007) |
| Religious belief | -0.020 (0.108) | -0.054 (0.131) | 0.024 (0.127) | 0.021 (0.112) |
| Political status | 0.257 (0.065)^***^ | 0.206 (0.098)^*^ | 0.128 (0.089) | 0.295 (0.066)^***^ |
| Household registration | 0.112 (0.052)^*^ | -0.142 (0.095) | 0.028 (0.070) | 0.005 (0.055) |
| Place of residence |  |  | 0.039 (0.070) | 0.057 (0.050) |
| Employment status | 0.066 (0.058) | -0.072 (0.068) | 0.002 (0.074) | -0.032 (0.052) |
| Marital status | 0.443 (0.072)^***^ | 0.399 (0.089)^***^ | 0.440 (0.099)^***^ | 0.442 (0.066)^***^ |
| Personal income | -0.009 (0.008) | 0.024 (0.009)^**^ | 0.004 (0.010) | 0.006 (0.007) |
| Socioeconomic status | 0.486 (0.028)^***^ | 0.472 (0.029)^***^ | 0.515 (0.035)^***^ | 0.466 (0.024)^***^ |
| Family size | 0.043 (0.014)^**^ | 0.039 (0.013)^**^ | 0.025 (0.016) | 0.045 (0.012)^***^ |
| Housing units | 0.057 (0.036) | 0.025 (0.056) | 0.003 (0.047) | 0.052 (0.039) |
| Per capita household income | 0.077 (0.023)^**^ | 0.059 (0.020)^**^ | 0.061 (0.025)^*^ | 0.075 (0.019)^***^ |
| Year fixed effect | Yes | Yes | Yes | Yes |
| Provincial fixed effect | Yes | Yes | Yes | Yes |
| N | 9,874 | 7,544 | 6,037 | 11,381 |
| Pseudo *R*^2^ | 0.074 | 0.097 | 0.076 | 0.085 |

Note: +, *, **, and *** indicate *P* <0.1, *P* <0.05, *P* <0.01, and *P* <0.001 respectively. Yes means the corresponding variables are controlled in the regression.
